# Genome sequence of the endophytic biocontrol and plant growth-promoting *Serratia marcescens* strain Sm25P5 from wheat seed

**DOI:** 10.1128/mra.00549-26

**Published:** 2026-08-11

**Authors:** Julfikar Ali, Lamya Muazzeda Medha, Turjoy Chandra Karmokar, Soharth Hasnat, Dipali Rani Gupta, Tofazzal Islam

**Affiliations:** 1 Institute of Biotechnology and Genetic Engineering (IBGE), Gazipur Agricultural University (GAU) 198780, Gazipur, Bangladesh; Indiana University Bloomington, Bloomington, Indiana, USA

**Keywords:** seed endophyte, antifungal, growth promotion, Oxford Nanopore sequencing (ONT), Plant probiotic

## Abstract

We report the whole genome of *Serratia marcescens* strain Sm25P5, an endophyte isolated from blast-resistant wheat seed. The 5.1-Mb genome harbors diverse putative biosynthetic gene associated with nutrient accumulation, mobilization, and antifungal activity, supporting its plant growth-promoting and biocontrol potential.

## ANNOUNCEMENT

*Serratia* species are gram-negative bacteria ubiquitous in natural and agricultural ecosystems. While some strains act as opportunistic pathogens (1, 2), many function as beneficial plant endophytes or epiphytes (3). These beneficial *Serratia* spp. possess plant growth-promoting (PGP) traits, such as nitrogen fixation, phosphate solubilization, siderophore production, and antifungal activity (3–6). *S. marcescens* strain Sm25P5 was isolated from the seeds of the blast-resistant wheat variety BARI Gom 33. The seeds were collected from an experimental field at Gazipur Agricultural University, Bangladesh (24.0362° N, 90.3959° E). For isolation, seeds were surface-sterilized with 80% ethanol (10 min), followed by 1% NaOCl (5 min), and rinsed three times with distilled water. Next, 1 g of pooled sample was homogenized in 9 mL of distilled water and cultured on a commercial nutrient broth agar (Oxoid) plate at 27 ± 2°C for 24 h to obtain single colonies. A single red colony was purified and designated Sm25P5.

The pure isolate was cultured in nutrient broth (Oxoid) for 24 h at 27 ± 2°C under shaking conditions (180 rpm). Genomic DNA was extracted from the 24-h culture using the GeneJET Genomic DNA Purification Kit (Thermo Fisher Scientific, USA) following the manufacturer’s protocol, and the concentration was quantified using a Qubit 4.0 fluorometer (Thermo Fisher Scientific, USA). Sequencing libraries were prepared using the ONT Ligation Sequencing Kit (SQK-LSK114.24) with the NEBNext companion module and Native Barcoding Kit 96 (v14) without DNA shearing or size selection. Sequencing was performed on a MinION with an R10.4.1 flow cell (7), and basecalling was done using Dorado v1.2.0 (SUP model). Raw read quality was assessed using NanoPlot v1.46.1 (8). The data set comprising 63,684 reads (mean length: 4,812 bp; *N*50: 7,521 bp) was filtered for quality (*Q* > 15) and length (>1,000 bp) using NanoFilt v2.8.0 (9). *De novo* assembly was performed with Flye v2.9.5 (10), and the resulting assembly was polished using Medaka v2.0.1 (11). Assembly completeness was evaluated with BUSCO v6.0.0 (12). The genome was annotated via the National Center for Biotechnology Information (NCBI) Prokaryotic Genome Annotation Pipeline (PGAP) v6.10 (13), with metabolic profiling performed using the RAST v2.0 server (14). Plasmid replicons and CRISPR arrays were identified using PlasmidFinder v2.0.1 (15) and CRISPRCasFinder v2.0.3 (16), respectively.

The genome of *S. marcescens* Sm25P5 consists of a single circular chromosome of 5,093,544 bp with a guanine-cytosine (GC) content of 59.5% (Table 1) . PGAP annotation identified 4,867 total genes, including 4,739 protein-coding sequences (CDSs), 90 tRNAs, and 22 rRNA. PGAP analysis identified putative PGP genes involved in nitrogen assimilation (*nifJ*, *narK*, *narX*), phosphate solubilization (*pqqC-F*), potassium regulation (*trkA*, *kdpA*), and sulfur mineralization (*cysA*, *tauA*) and putative biocontrol-related genes, including chitinases (*chiABC*), prodigiosin (*pigCI*), and acetoin/2,3-butanediol pathways (*budAC*), suggesting potential roles in plant growth promotion and biocontrol (Table 1) (3–6, 17). PathogenFinder v2.0 (18) predicted strain Sm25P5 to be nonpathogenic (score: 0.4), suggesting a low risk for agricultural application. Default parameters were employed for all bioinformatic tools. These genomic features indicate the strain as a candidate for further evaluation as a biofertilizer and biopesticide.

**TABLE 1:** Genomic features of *Serratia marcescens* strain Sm25P5 isolated from BARI Gom 33.

Genome feature	Sm25P5
GenBank accession	JBTOFK000000000
BioSample accession	SAMN53476057
SRA	SRX32351100
Assembled genome size (bp)	5,093,544
GC %	59.5
Coverage	53×
Genome completeness (%)	99.8 (BUSCO)
CheckM contamination (%)	0
*N*50 value (bp)	5,093,544
*N*90 value (bp)	5,093,544
Number of contigs	1
Genes (total)	4,867
CDSs (total)	4,739
Genes (coding)	4,715
CDSs (with protein)	4,715
Genes (RNA)	128
rRNAs	8, 7, 7 (5S, 16S, 23S)
Complete rRNAs	8, 7, 7 (5S, 16S, 23S)
tRNAs	90
ncRNAs	16
Pseudo genes (total)	24
CDSs (without protein)	24
Pseudo genes (ambiguous residues)	0 of 24
Pseudo genes (frameshifted)	12 of 24
Pseudo genes (incomplete)	17 of 24
Pseudo genes (internal stop)	3 of 24
Pseudo genes (multiple problems)	8 of 24
CRISPR arrays	0
Plasmid replicon	0

## Data Availability

The genome sequence has been deposited in GenBank under accession JBTOFK000000000 associated with BioProject PRJNA1055760 (Mining Bangladesh Biogold), BioSample SAMN54567177, SRA SRX32351100, and 16S rRNA sequence PV290559.
